# Single Portal Segmental Labral Reconstruction of the Hip

**DOI:** 10.1016/j.eats.2023.02.006

**Published:** 2023-04-24

**Authors:** Matthew J. Hartwell, Daniel B. Goldberg, Samuel G. Moulton, Stephanie E. Wong, Alan L. Zhang

**Affiliations:** Department of Orthopaedic Surgery, University of California–San Francisco, San Francisco, California, USA

## Abstract

Many techniques have been described for reconstruction of the acetabular labrum, but the procedure is known to be technically rigorous leading to lengthy procedure times and traction times. Increasing efficiency of the procedure with respect to graft preparation and delivery remain areas for potential improvement. We describe a simplified procedure for arthroscopic segmental labral reconstruction using peroneus longus allograft and a single working portal to shuttle the graft into the joint via suture anchors placed at the terminal extents of the graft defect. This method allows for efficient preparation, placement and fixation of the graft that can be completed in under 15 minutes.

## Introduction

The acetabular labrum plays a vital role in the kinematics of the hip, serving to stabilize the femoral head, provide an increased load bearing surface and maintain a suction seal for the joint.^1^, ^2^, ^3^ Disruption of the acetabular labrum can lead to increased contact pressure within the joint as well as significant pain and dysfunction for the patient.^4^^,^^5^ Multiple studies have demonstrated the efficacy of arthroscopic repair in the treatment of acetabular labral tears, however not all tear patterns or labral tissues are amenable to repair.^6^^,^^7^ In these cases, labral reconstruction may be considered.^8^^,^^9^ Current indications for labral reconstruction include ossification of the labrum, irreparable labral tears and iatrogenic loss of labral tissue.^6^^,^^10^ Hypoplastic labral tissue (<2-4mm) has also been cited as a possible indication for reconstruction due to inferior clinical results with repair alone.^8^^,^^11^ Recent literature has shown improved clinical outcomes with labral reconstruction and outcomes that rival those of primary repair in patients with moderate to severe labral damage.^9^^,^^12^, ^13^, ^14^ With expanding indications and improved patient outcomes, acetabular labral reconstruction has become increasingly utilized.

Although increasing in popularity, arthroscopic labral reconstruction is still a relatively new procedure, with the vast majority of described techniques being published in the last decade. There are many different approaches, and these techniques typically involve either the creation of a third arthroscopic portal and/or the use of various complex intra-articular suture management techniques to secure the graft. For example, the knotless pull-through technique published by Perets et al. requires the creation of a distal lateral anterior portal (DLAP) in order to introduce the graft into the joint.^15^ The technique also calls for the placement of all anchors prior to graft introduction, requiring the surgeon to execute meticulous suture management and additional passing. The “Kite technique” published by Bhatia et al. also requires the addition of an accessory working DALA portal.^16^ In this procedure all intermediate sutures are placed in the graft prior to delivery to be later fixed through a knotless anchor. This may require intricate intra-articular suture passing as all repair sutures are all brought into the joint with the labral graft. For other techniques that have not utilized an accessory working portal, reports describe passing suture within the joint and through a free floating end of the graft (or a previously placed stay suture) in order to secure the posterolateral aspect of the graft.^17^^,^^18^ Due to these remaining complexities within the available procedures, we believe there is a need for a simplified version of the arthroscopic labral reconstruction.

As arthroscopic labral reconstruction is known to be a technically challenging procedure, the goal of this article is to present a simplified and reproducible technique for acetabular labral reconstruction that minimizes procedural steps, thus reducing operative time and traction time which is important for reducing risk for complications.^19^ Our technique utilizes a single-working portal to shuttle and dock the graft followed by sequential anchor placement similar to standard labral repair. This eliminates the need for accessory working portals or multi-anchor intra-articular suture passing during arthroscopic labral reconstruction.

### Surgical Technique

Video 1 demonstrates our technique for arthroscopic segmental labral reconstruction in the hip. The procedure starts by placing the patient supine for hip arthroscopy. We prefer a post-free hip distraction system. The patient is then prepped, draped, and the limb placed in neutral flexion and rotation. An air arthrogram is performed to decompress the negative pressure and break the suction seal within the joint to allow for adequate distraction. An anterolateral portal (ALP) is then established under fluoroscopic guidance and a 70° arthroscope is introduced into the joint. A modified mid-anterior portal (MAP) is established under direct arthroscopic visualization. Periportal capsulotomies of the ALP and MAP are performed, as previously described.^20^^,^^21^ An 8 x 90-mm disposable plastic cannula (Smith & Nephew, Andover, MA) is then placed in the MAP, which is used as the primary working portal for the entire procedure.

**Fig 1:**
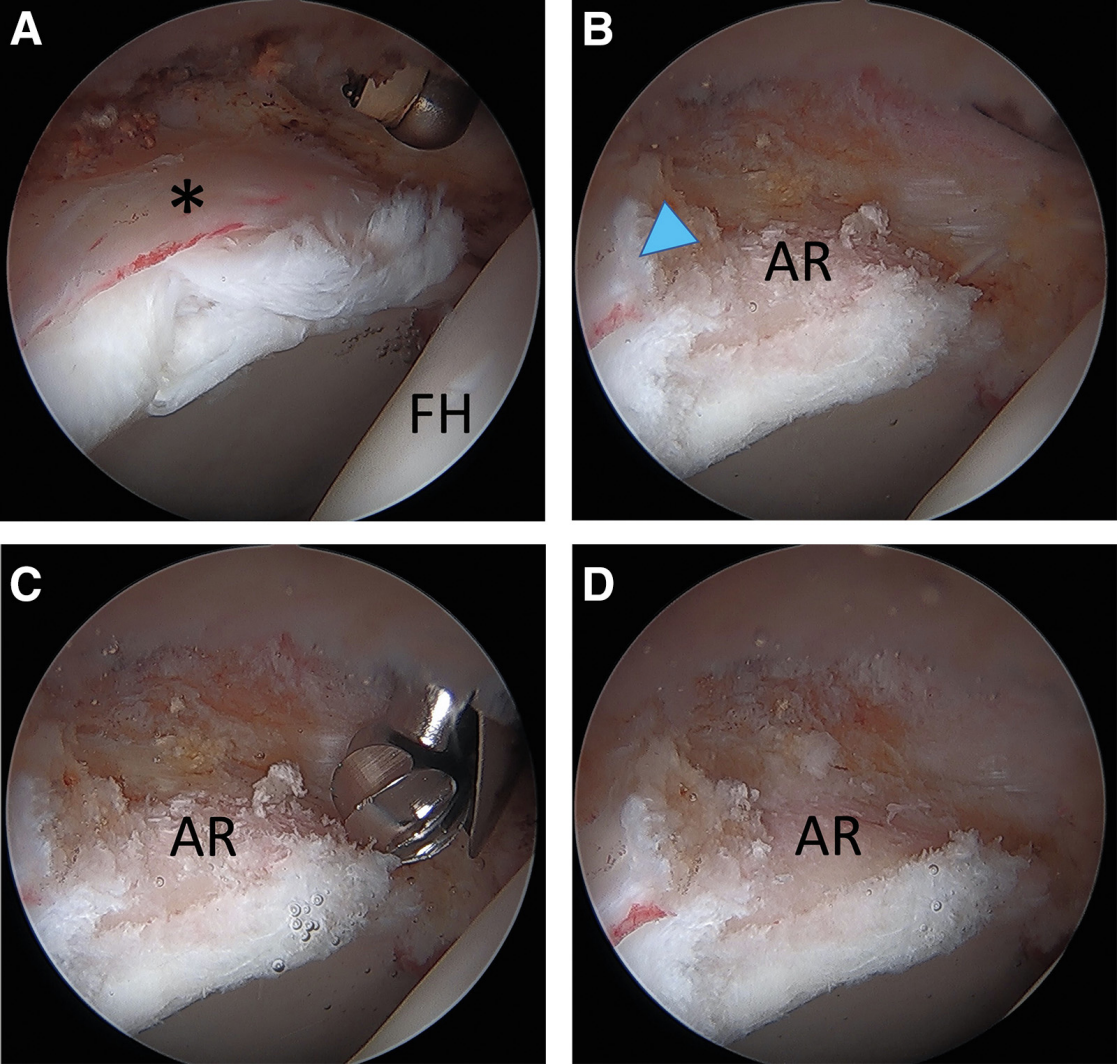
Arthroscopic images of a right hip in a supine position, viewing through an ALP. (A) A degenerative and diminutive labrum (black asterisk) is identified during diagnostic arthroscopy. (B) The labral tissue is debrided down to bone using the combination of an arthroscopic shaver and radiofrequency ablation until healthy labral tissue is reached (blue arrow). (C) A 5.5-mm arthroscopic round burr is used to perform an acetabuloplasty as needed for pincer resection and to create a level bony surface for graft incorporation. (D) The final prepared acetabular rim. (ALP, anterolateral portal; FH, femoral head; AR, acetabular rim).

Diagnostic arthroscopy is first performed to evaluate the viability of the labral tissue. In this case, a degenerative and diminutive labrum was identified (Fig 1A), and a reconstruction was elected given the unrepairable nature of the labrum. Next, a thorough debridement of the damaged labral tissue is performed until the edges of healthy labral tissue is reached (Fig 1B). A 5.5 mm arthroscopic round burr (Stryker, Kalamazoo, MI) is used to perform an acetabuloplasty as needed for pincer resection and to create a level bony surface for graft incorporation (Fig 1C). The acetabular rim is then ready for reconstruction (Fig 1D).

**Fig 2:**
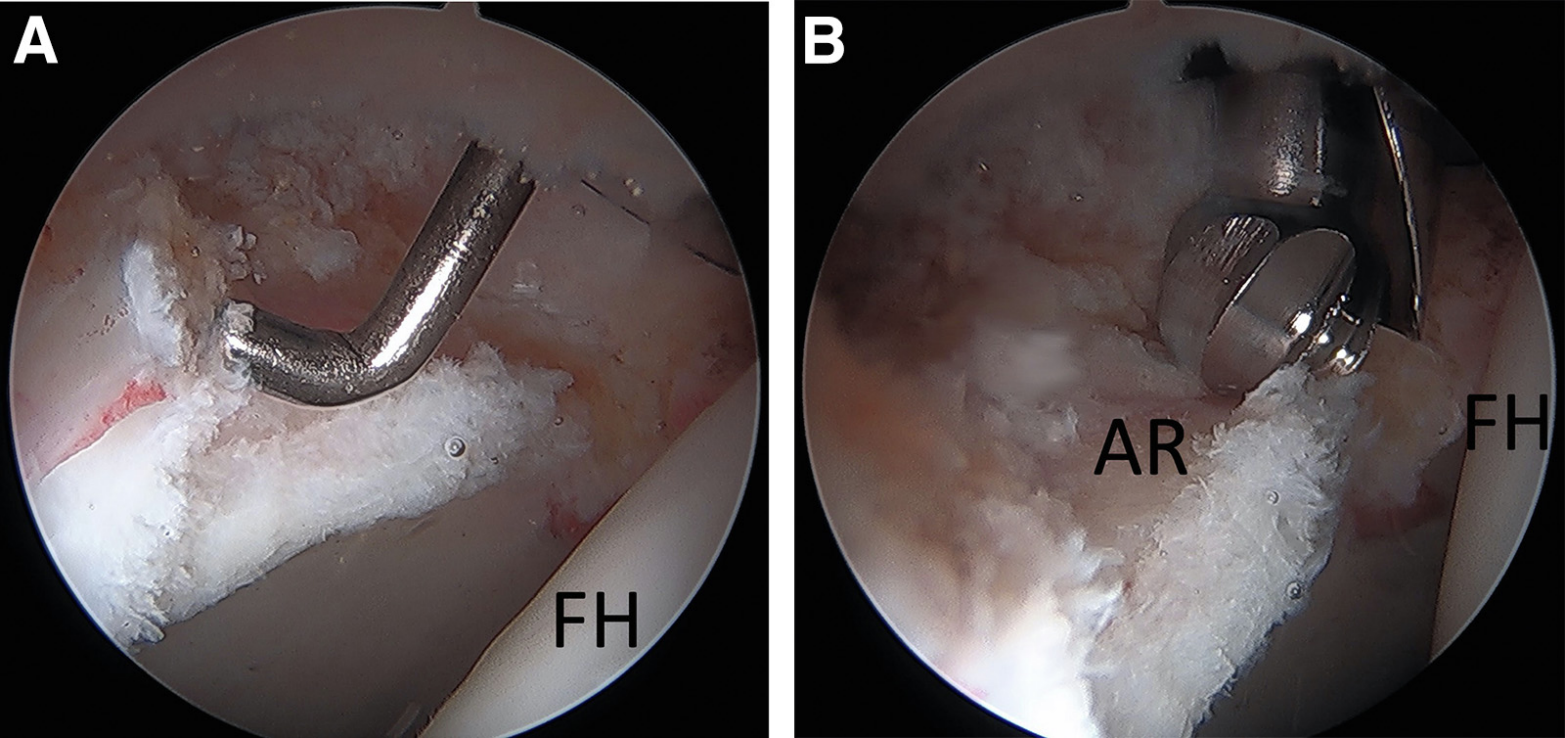
The size of the labral defect is determined, which can be accomplished with various methods. (A) The tip of an arthroscopic probe (3 mm width) or (B) the tip of a round arthroscopic burr (5.5 mm with) can be used to estimate the size of the defect. (FH, femoral head; AR, acetabular rim).

**Fig 3:**
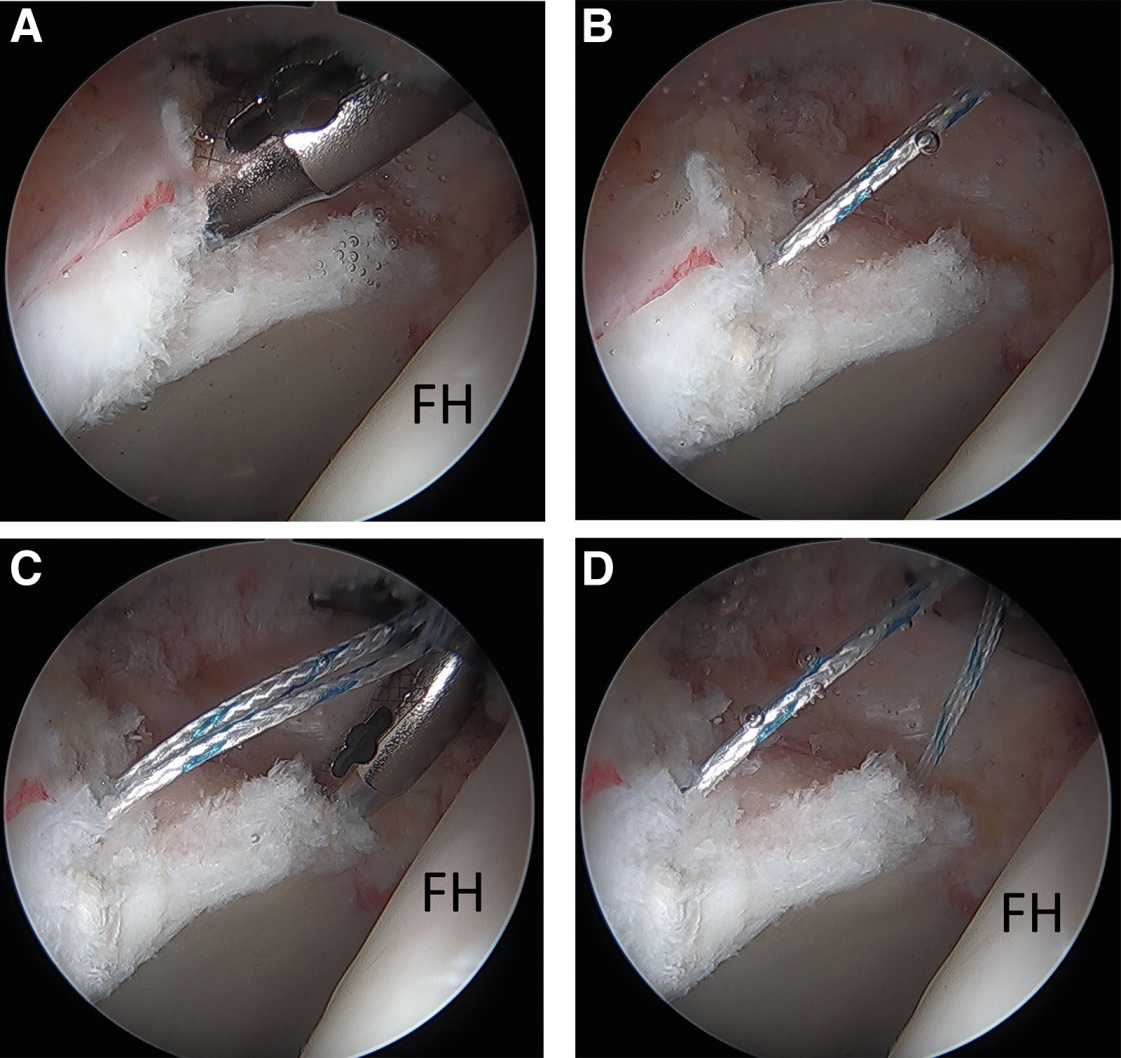
Two single-loaded suture anchors are placed through the mid-anterior portal (MAP) in the most anterior and posterior portions of the defect. Starting with the posterior anchor, a guide is used to drill for the posterior anchor (A) and an anchor is placed (B). The sutures are kept within the cannula but clamped and held to the side while the second anchor is placed. The guide is then placed at the most anterior portion of the defect (C) and a second anchor is placed (D). (MAP, mid-anterior portal; FH, femoral head).

Once ready to proceed with labral reconstruction, we begin by assessing the size of the defect. Multiple methods are available for arthroscopic measurement; we prefer using the end of an arthroscopic probe (Fig 2A), which has a width of 3 mm, as well as doubling checking with the tip of an arthroscopic burr (Fig 2B), which has a width of 5.5 mm. An arthroscopic ruler can also be used to measure this distance. Next, a single-loaded flexible PEEK anchor (NanoTack Flex; Stryker, Kalamazoo, MI) is inserted through the MAP and placed at the posterior (Fig 3A, 3B) edge of the labral defect immediately adjacent to the remaining intact labrum using a 25-degree curved guide. The sutures from the first anchor are kept in the cannula but tagged with a clamp and held to the side of the cannula while a second anchor is placed through the same working portal/cannula at the anterior edge of the labral defect (Fig 3C, 3D). It is important to keep sutures from each anchor separated within the 8mm cannula.

**Fig 4:**
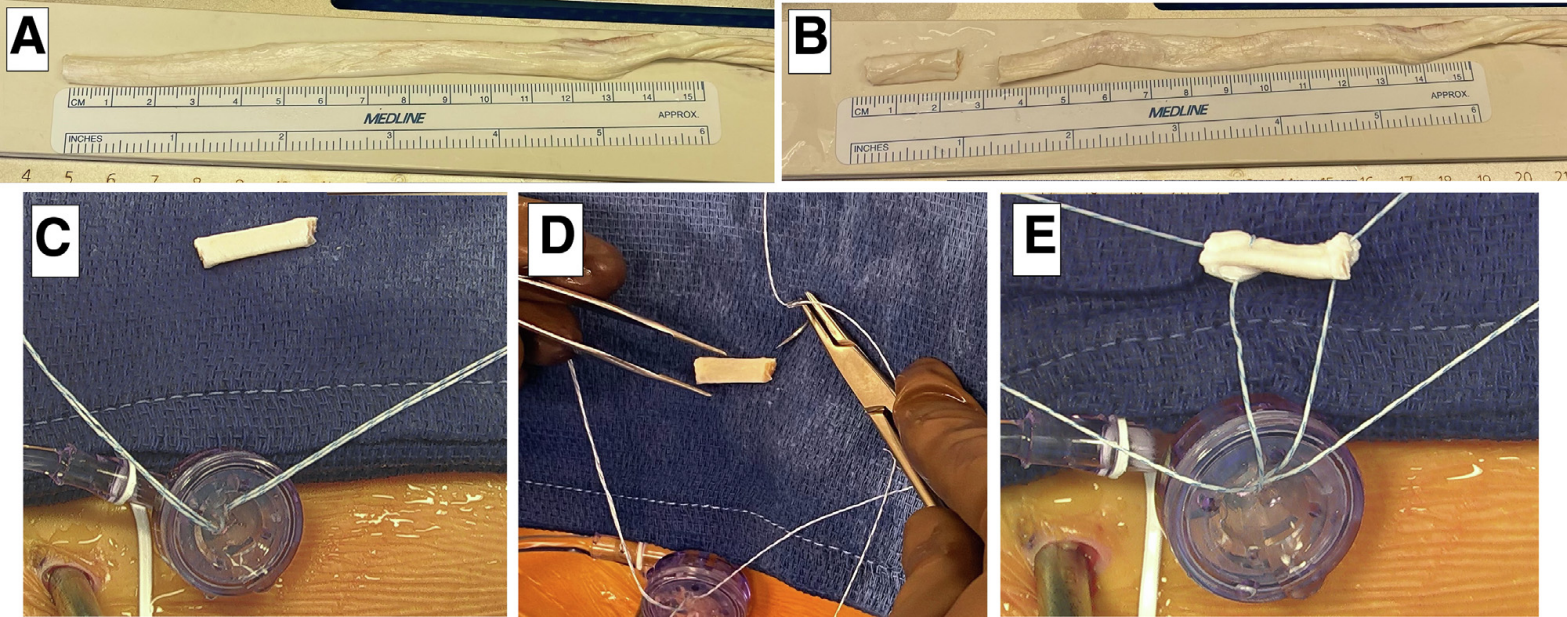
Preparation of the peroneus longus allograft, 5-6 mm in diameter. (A) The graft is thawed, and the tubular/triangular distal end of the graft is identified, which will be used for the reconstruction. (B) A ruler is used to cut the graft to the desired length of the defect. (C) The graft is then brought over and placed on a blue towel on this patient’s right thigh next to the mid-anterior portal and the suture limbs from each anchor are separated from one another. (D) One suture limb from each anchor is then shortened to half the length of the other end of the suture coming out of the cannula, and the longer limb end from each anchor is sutured into the edge of the graft, in a figure-of-8 fashion, leaving enough of a tail coming out of the graft that can be used later for tying. (E) The graft is then ready for shuttling into the joint.

**Fig 5:**
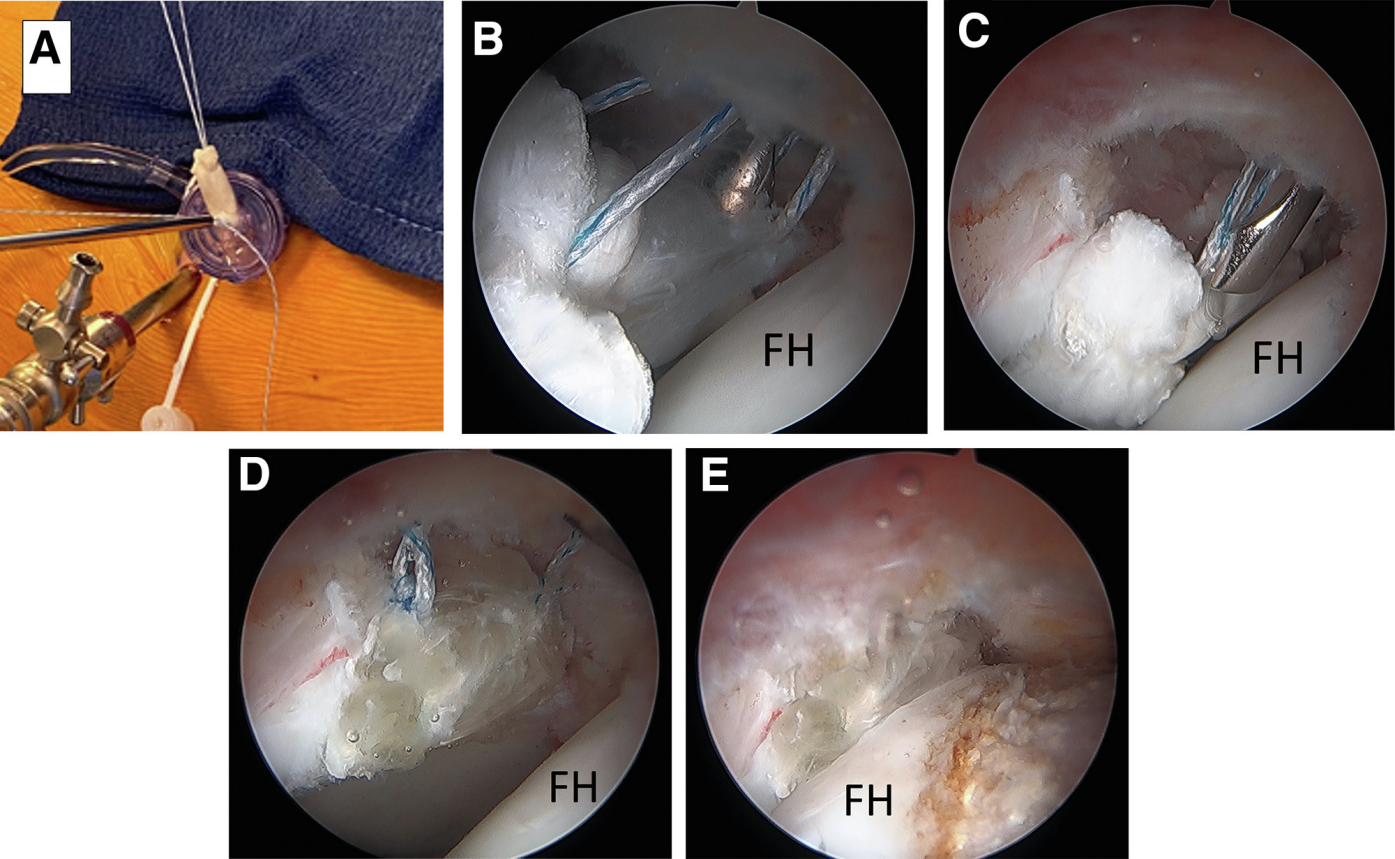
Clinical and arthroscopic images of a patient’s right hip demonstrating graft shuttling into the joint. (A) Starting with the sutures in the anterior anchor, a single half hitch, using the tagged suture coming out of the graft, is first thrown onto the shortened post suture. The post is then pulled up to deliver the graft into the joint. A knot pusher through the post can help push the graft down the cannula and to the acetabular rim. The shortened suture limb in the posterior anchor (the post) is simultaneously pulled to remove slack as the graft is shuttled. (B) The posts from both anchors can then be pulled to seat the graft into the defect and a series of 5 alternating half hitches are tied and cut. (C) The graft is the tied down posteriorly. (D) Additional suture anchors can be placed in the central portion of the graft as needed for further fixation of the construct. (E) Traction can then be released, which demonstrates restoration of the suction seal between the labrum and femoral head. (FH, femoral head).

The graft is then prepared, which can be performed by an assistant once the size of the labral defect has been determined. We prefer the use of a peroneus longus allograft that is 5-6 mm in diameter, similar in size to an intact and normal labrum. The tubular/triangular distal end of the graft will be used for the reconstruction (Fig 4A). A ruler is used to measure and cut the graft to the same length as the labral defect (Fig 4B). The graft requires no further preparation and is then placed on a blue towel on the patient’s thigh next to the MAP cannula so the sutures from the two previously placed anchors can be used to tag the ends of the graft for shuttling (Fig 4C). Prior to passing the sutures into the graft, one suture limb from each anchor is shortened to half the length of the other end of the suture coming out of the cannula. The longer strand will be used to tag the graft; ensure enough of a tail is remaining out of the graft after tagging so it can be used for tying later. The strand going through the graft will be the non-post and the shortened free strand will be the post. A tapered (non-cutting) free needle is used to pass the longer strands of the suture limbs through the graft (Fig 4D). These are placed near the ends of the graft in a tight figure-of-8 fashion (Fig 4E). The sutures from the posterior terminal anchor will be passed through the end of the graft that is docked posteriorly and the anterior terminal anchor sutures will be passed through the end of the graft docking anteriorly. The graft is then ready for shuttling into the joint. The anterior part of the graft is passed into the joint and docked to the anterior anchor first. A half-hitch from the tagged suture coming out of the anterior end of the graft (non-post) is first thrown onto the post suture, and the post is then pulled up to deliver the graft into the cannula and into the joint. A knot pusher through the post can be used to help push the graft down the cannula to the anterior anchor on the acetabular rim (Fig 5A). Slack is pulled out of the sutures in the posterior anchor by pulling on the post of the posterior anchor (the shortened free strand) while shuttling the graft. Once the anterior aspect of the graft is visualized to be fully docked to the anterior anchor, the post of the posterior anchor is pulled tight to deliver the posterior part of the graft to the posterior anchor. Pulling on both post limbs tight will seat the graft into the defect. Next the sutures from the anterior anchor are firmly tied down to the graft with 5 alternating half hitches and cut (Fig 5B). The sutures in the posterior anchor are then tied down with 5 alternating half hitches and cut (Fig 5C). Now that the labral reconstruction is docked and secured at the ends of the defect, additional anchors can then be sequentially placed as needed in the center portion between the anterior and posterior terminal anchors to secure the remainder of the construct, using loop suture configurations as performed for routine labral repairs. This eliminates the need for simultaneous suture management of multiple anchors. Excess graft can be trimmed using a shaver or radiofrequency ablation device (Fig 5D). Traction can then be released to assess the final construct and suction seal, which can be seen here following a subsequent femoroplasty (Fig 5E).

**Fig 6:**
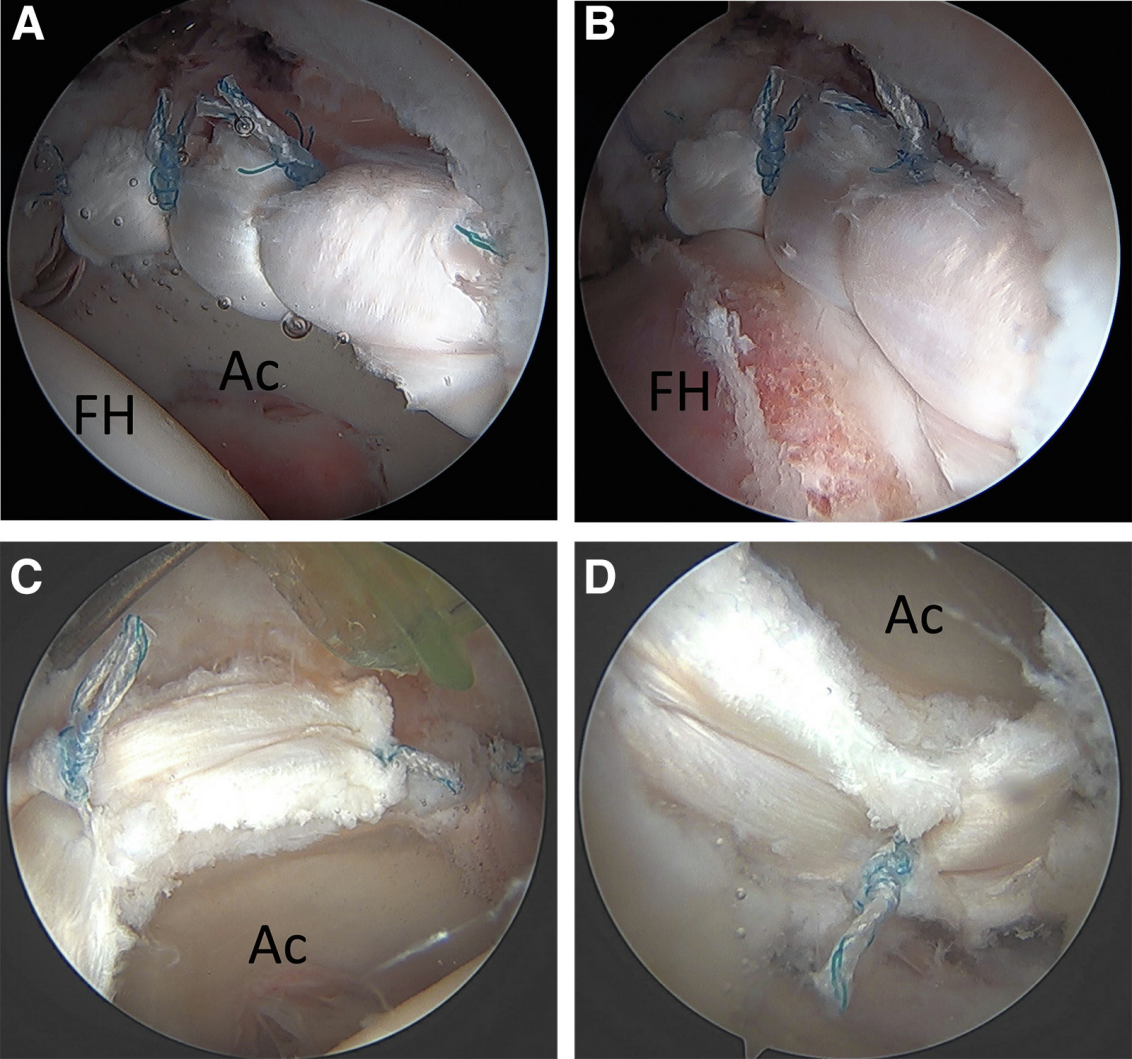
Examples of using this single working portal technique for varying defect lengths and locations around the acetabulum. (A) An example of a longer segmental labral reconstruction in a left hip using four suture anchors and (B) the restoration of the suction seal after release of traction. (C) An example of a more posteriorly based reconstruction in a right hip using three suture anchors, viewing the more anterior portion of the reconstruction and (D) posterior portion of the reconstruction. (FH, femoral head; Ac, acetabulum).

This technique for segmental labral reconstruction is performed through an 8x90mm disposable cannula in the MAP without switching working portals and can be efficiently completed in under 15 minutes. This technique can also be utilized for varying defect lengths and a variety of locations around the acetabulum. Longer segmental graft reconstructions can be accomplished, as seen in this example of a reconstruction that used four suture anchors (Fig 6A, 6B). It can also be used for more posteriorly based reconstructions, as seen in this reconstruction of a posterior labral defect in which the single working portal was placed in the ALP and the MAP was used for the viewing portal (Fig 6C, 6D).

Advantages and disadvantages (Table 1) as well as pearls and potential pitfalls (Table 2) of this technique are summarized.

**Table 1:** Advantages and Disadvantages

Advantages	Disadvantages
• Single working portal • Can address both anterior and posterior acetabular defects without the creation of additional portals • Efficient and simplified method for graft preparation—using peroneus longus allograft only requires cutting the graft to the length desired • Reduced traction times (technique can be performed in 15 minutes) • Eliminates need for suture management for multianchors placed simultaneously prior to graft docking	• Accurate measurement of labral defect length is needed • Far anterior and far posterior defects may be difficult for anchor placement through a single working portal • Large labral reconstructions (>50mm length) and pan-labral reconstructions are difficult to perform with this technique as anchor placement near the transverse acetabular ligament is difficult through a single midanterior portal

**Table 2:** Pearls and Pitfalls

Pearls	Pitfalls
• Fully debride poor-quality labral tissue and perform acetabuloplasty to create flat landing surface on acetabulum prior to reconstruction • Accurately determine and check graft length through different techniques • Ensure sutures from each anchor at the ends of the reconstruction do not twist or tangle with each other inside the disposable cannula • Pass shuttling sutures from anchors through the ends of the graft near the edge to avoid excess tissue • Working portal can be used through midanterior portal for anterior-superior defects or anterolateral portal for posterior defects • Place anchors near the rim of the acetabulum/cartilage junction to create good suction seal with the graft	• Sutures from two anchors twist around each other inside of the cannula and graft becomes twisted as it is delivered into the joint • Graft not cut to the exact length of the defect, resulting in bunching of the graft if too long or insufficient coverage if too short • Suture anchors are placed too medial to the acetabular rim and the labral reconstruction graft is everted, losing suction seal effect

## Discussion

Arthroscopic labral reconstruction is well-known to be a challenging procedure, as multiple techniques published over the last decade involve either the creation of a third arthroscopic working portal and/or complex suture management techniques to secure the graft. The “Kite Technique” is one method of labral reconstruction that requires both an additional portal and intricate suture management.^16^ In this technique the graft is delivered through the DALA portal by tensioning shutting sutures that exit the AL portal and knots are then tied through the MA portal. This necessitates that the surgeon work through all three portals and pass suture between the portals multiple times during the operation. The “knotless pull-through” technique also requires the placement of an additional accessory portal and the surgeon must place multiple suture anchors that will be used to secure the mid-body of the graft prior to graft delivery. The associated sutures must then be passed, stored, and managed during graft delivery.

In contrast to these techniques, we have found that by using the suture-anchor based shuttling method in our current technique, the graft can be reliably delivered into the central compartment through a single working portal. By shuttling the graft via suture anchors placed at the terminal edges of the labral defect, the need for complex suture passing and management is minimized, thus simplifying the delivery of the graft into the joint. After the terminal anchors are tied, additional anchors can then be placed in between and tied one at a time similar to a simple labral repair, eliminating excessive suture passing and opportunities for entanglement. In addition, the single working portal technique presented in this article allows the surgeon to address both anterior and posterior labral defects by utilizing the MA or the AL portal respectively, as the primary working portal.

In our review of the literature, we found previously published techniques for labral reconstruction using the standard AL and MA portal. However, the strategies for graft delivery and fixation varied substantially from the method presented here. Park et al. described a two-portal technique using quadriceps tendon autograft.^18^ In this technique, the most anterior and posterior anchors are placed prior to harvest. The graft is shuttled into the hip along a suture from the most anterior anchor via the MA portal using a knot pusher. The surgeon must then pass suture from the posterior anchor through a previously placed control-stich while one limb of the graft floats freely in the central compartment. Chahla et al. also published a two-portal technique in which the graft is delivered and secured in a near identical fashion to the Park et al. method, but using iliotibial band autograft.^17^ Both techniques require intra-articular passage of suture through a partially anchored graft, adding time and complexity to the procedure in addition to autograft harvest. Our technique allows for fixation of both ends of the graft to the anterior and posterior anchors as the graft is passed into the joint without the need to pass suture through a free floating graft end. Further, our use of peroneus longus allograft allows for minimal graft preparation time, further reducing the overall procedure and traction time.

In conclusion, we present a technique for arthroscopic segmental acetabular labral reconstruction using a single working portal and peroneus longus allograft that can be completed in under 15 minutes. This simplified technique improves the efficiency of the procedure by delivering both ends of the graft through a single working portal with anchors placed at the anterior and posterior extents of the labral defect followed by routine labral repair between the terminal anchors after graft docking.

## Supplementary Data

ICMJE author disclosure forms

Video 1 This technique demonstrates segmental reconstruction of an acetabular labrum with the use of a single working portal in a right hip. Two standard anterolateral (AL) and modified midanterior (MA) portals are established with the AL used as the viewing portal and the MA the single working portal. A peroneus longus allograft, 5-6 mm in diameter is the preferred graft choice.
